# “Hit the missing stimulus”. A simultaneous EEG-fMRI study to localize the generators of endogenous ERPs in an omitted target paradigm

**DOI:** 10.1038/s41598-019-39812-z

**Published:** 2019-03-06

**Authors:** Aldo Ragazzoni, Francesco Di Russo, Serena Fabbri, Ilaria Pesaresi, Andrea Di Rollo, Rinaldo Livio Perri, Davide Barloscio, Tommaso Bocci, Mirco Cosottini, Ferdinando Sartucci

**Affiliations:** 1 PAS Foundation, Scandicci, Italy; 2 Department of Movement, Human and Health Sciences, University of Rome “Foro Italico”, Rome, Italy; 3 Neuroradiology Unit, A.O.U.P., Pisa, Italy; 4 Department of Translational Research and New Technologies in Medicine and Surgery, University of Pisa, Pisa, Italy; 5 Department of Clinical and Experimental Medicine, Unit of Neurophysiopathology, Pisa University Medical School, Pisa, Italy; 6 CNR, Neuroscience Institute, Pisa, Italy; 7 “Aldo Ravelli” Center for Neurotechnology and Experiental Brain Therapeutics, Department of Health Sciences, University of Milan & ASST Santi Paolo e Carlo, Milan, Italy

## Abstract

Event-Related Potentials (ERPs) occurring independently from any stimulus are purely *endogenous* (*emitted potentials*) and their neural generators can be unequivocally linked with cognitive processes. In the present study, the subjects performed two similar visual counting tasks: a standard two-stimulus oddball, and an omitted-target oddball task, characterized by the physical absence of the target stimulus. Our investigation aimed at localizing the neural sources of the scalp-recorded *endogenous/emitted* ERPs. To optimize the source localization, the high temporal resolution of electrophysiology was combined with the fine spatial information provided by the simultaneous recording of functional magnetic resonance (fMRI). Both tasks identified two *endogenous* ERP components in the 300 to 520 ms interval. An earlier component, pP2, showed a bilateral generator in the anterior Insula. A later P3 component (P3b) was generated bilaterally in the temporal-parietal junction, the premotor and motor area and the anterior intraparietal sulcus (this latter one only in the standard oddball). Anticipatory slow waves (beginning 900 to 500 ms pre-stimulus), also of *endogenous* nature, were produced by the inferior and middle frontal gyrus and the supplementary and cingulate motor areas. Our protocol disentangled pre- from post-stimulus fMRI activations and provided original clues to the psychophysiological interpretation of emitted/endogenous ERPs.

## Introduction

The *endogenous and exogenous* dual nature of event related potentials (ERPs) has been highlighted since the early days of their discovery^1^. As opposed to early latency *exogenous* components which are primarily determined by the physical features of the eliciting stimulus, *endogenous* components have longer onset latencies (>100 ms) and are assumed to reflect successive stages of information processing activated by the significance of the stimulus rather than by its physical features. Accordingly, *exogenous* ERPs mainly emerge from a “bottom-up” flow of sensory input whereas *endogenous* ERPs mainly express a “top-down” modulation of complex neurodynamic. A proof-of-concept study has been recently provided for differentiating in humans sensory-specific macrosignals encoding sensory information from supramodal signals reflecting a neural process irrespective of specific sensory or motor requirements, leading to perceptual decisions^2^. Owing to their unique sensitivity to cognitive factors, *endogenous* potentials are also referred to as “cognitive” ERPs^3^. Over the past 50 years, ERPs obtained with different stimuli and task paradigms have been extensively used for studying a multitude of cognitive processes such as attention, memory, language and executive functions (for reviews see^4–6^). In addition, they have been increasingly employed in diagnostic investigations of neurological and psychiatric disorders (for reviews see^4,7^). The P3 (also known as P300 or P3b or late positive component, LPC) is the most studied ERP component and defined as a supramodal, positive component peaking from 300 to 800 ms at medial centro-parietal areas whenever a task-relevant stimulus is detected. The P3 is commonly elicited in the so-called “oddball” paradigm in which subjects have to distinguish the rare target stimuli randomly embedded in a stream of repetitive frequent standard stimuli. It has been associated with many cognitive operations ranging from selective attention to working memory, from stimulus categorization to response selection, task closure or even inhibition^5^. The P3 has been also proposed as a neurophysiological signature of conscious perception^8,9^; with a divergent interpretation summarized by^10^. According to the “context updating theory” (Donchin & Coles^11^), the core cognitive operation reflected by P3 is the updating of some model of the environment whenever a conflict arises between new information and expectations. The P3 is therefore a strategic ERP component associated with a meta-control function operating on “priorities, biases and probabilities”^11^ (for a recent overview^12^). Of major importance in order to outline the peculiar psychophysiological features of P3 is the identification of its neural generators. A number of studies conducted with intracranial recordings in neurosurgical patients^5,13–16^, electroencephalogram (EEG) or magnetoencephalography (MEG) in patients with focal brain lesions^16–18^ or functional MRI^19–25^ demonstrated that P3 can be collected from many cortical and subcortical locations. Namely, P3 generators have been found in the superior temporal sulcus, inferior parietal cortex and intraparietal sulcus, lateral and medial prefrontal cortex, the insula, hippocampus, amygdala, thalamus and motor cortex. Such a widespread distribution of sources in the brain suggests that the P3 recorded from the scalp results from multiple, partially independent generators belonging to large-scale brain networks active during target processing^26,27^. However, despite the numerous investigations, the neural sources of the P3 remain somehow elusive and the results provided by the intracranial approach are partly different from those emerging from the fMRI analysis. We sought to investigate the neural generators of endogenous ERPs (including the P3) by the simultaneous recording of EEG and fMRI during a visual oddball paradigm, capitalizing on the high temporal resolution of EEG (in the range of milliseconds) and the excellent spatial sampling (in the range of millimeters) provided by fMRI^23,28–33^. An estimation of time-course of the cerebral generators of ERP components was performed by applying a discrete multiple source analysis to the EEG guided (seeded) by fMRI data. In order to solve the inverse problem of ERP source localization, the fMRI activation spots were used to constrain the number and spatial location of EEG dipolar sources (fMRI-guided seeding model^24,25,34–36^.

A usual confounding factor when studying the neural generators of cognitive ERPs, is that *exogenous* components can partly overlap in space and time with *endogenous* potentials, resulting in spurious results^37^. However, ERPs –mostly the P3- can be generated in an oddball paradigm in which the target stimulus is represented by the omission of the stimulus itself (omitted stimulus paradigm^1,38–53^). ERPs occurring independently of any specific evoking sensory event are purely *endogenous* (*emitted potentials*) and therefore best suited for the investigation of neural generators univocally linked with cognitive components. In the present ERP-fMRI study, the subjects performed two simple tasks: a two-stimulus standard visual oddball and a similar, omitted-target oddball. The main goal of the present investigation was to localize the brain sources of the scalp-recorded ERPs and to compare the results obtained by these two oddball paradigms. Our assumption was that the source analysis of ERPs obtained with the omitted-target oddball task, selectively eliciting endogenous components, allows an accurate and reliable identification of the brain’ functional anatomy subserving target processing.

As the BOLD signal can encompass both *pre-* and post*-stimulus* activities due to its long time constant. *Pre-stimulus* ERP activity was also analyzed to check out whether part of fMRI activations could be related to anticipatory slow waves as the Bereitschaftspotential (BP) associated with response readiness in premotor cortex^54^ and the prefrontal negativity (pN) associated with cognitive preparation intended as proactive attention and inhibition in the prefrontal cortex^55^. This was a crucial step to identify which sources were linked to specific ERP components either preceding or following the stimuli.

Further, the investigation included the *exogenous* (obligatory) visual ERPs (i.e., the posterior P1, N1 and P2 associated with visual processing in occipital areas and the prefrontal N1, P1 and P2 (pN1, pP1 and pP2) associated with sensorial and sensory-motor awareness (the pN1 and pP1) and with stimulus-response mapping (the pP2) within the anterior insular cortex^35,56^.

Predictions about the present standard-oddball task were to replicate the findings of our previous studies exploring ERP spatiotemporal mapping with a fMRI-seeded dipole method using different tasks (equal target/non-target probability go/no-go tasks) confirming the presence and the intracranial origin of the recently described prefrontal and insular ERP components^35,57^. For the omitted-target task we expected to find a pattern of brain activations fully devoid of any contribution from sensory encoding signals and therefore including purely endogenous components as the P3 and possibly the pP2 at post-stimulus level, but also the proactive BP and pN.

## Material and Methods

### Participants

Thirteen healthy right-handed participants volunteered (5 females; mean age 26 years, range 22–29), recruited from an academic environment. All subjects had no history of neurological or psychiatric disorders; they were not using medications and had normal or corrected-to-normal vision. They were naïve to electrophysiological recordings. All participants gave an informed consent to participate in the study. Methods were carried out according to the rules of the University of Pisa Ethical Committee that approved all experimental protocols.

### Stimuli and task design

**Figure 1:**
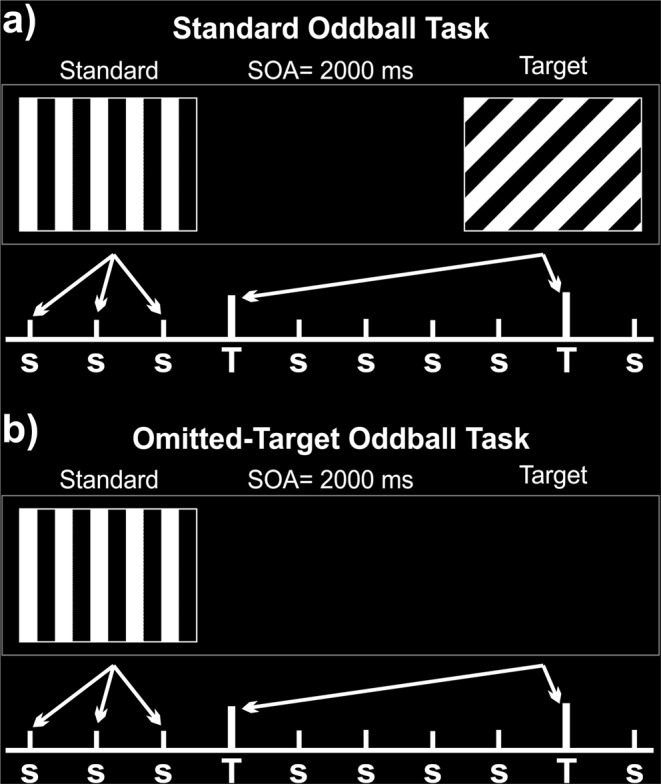
Schematic representation of the two experimental tasks: (**a**) Standard visual Oddball; (**b**) Omitted-Target Oddball. s: standard-frequent stimuli. T: target-rare stimuli. SOA: Stimulus-Onset Asynchrony.

fMRI and EEG data were acquired simultaneously in a single experimental session. The simultaneous recording of EEG and fMRI guaranteed that the cognitive states and the body position of the subjects were the same for the two techniques during the experimental session. The session included two distinct types of task, and both were administered in an active and a passive condition. The two tasks differed in the properties of the target stimuli: one task consisted of a two-stimulus visual oddball paradigm, in which rare stimuli (targets) were unpredictably intermingled with frequent non-target stimuli (*Oddball task* in Fig. 1a). Stimuli were delivered through MRI compatible fiber-optic goggles (VisuoStim-Resonance Technologies, Northridge, CA, U.S.A.). They consisted of square-wave gratings of black and white bars, with a spatial frequency of 0.5 cycle/degree and 200 ms duration, presented on a continuous black background either with a vertical orientation (Frequent non-target stimuli: probability 0.80) or tilted by 45 degrees (Rare target stimuli: probability 0.20). Visual stimulation was managed with Presentation (Neurobehavioral Systems, Berkeley, CA, U.S.A.). The stimulus-onset asynchrony (SOA) was set to 2 seconds. The other task (*Omitted Target task* in Fig. 1b) was similar in all details to the *Oddball task* except that the target stimulus in this series was represented by the omission of the visual stimulus (omitted target). To avoid any contamination from motor activities, a simple covert counting performance was required. In both tasks, the participants received instructions to mentally count the rare target and report the total at the end of the run (active tasks). Adequate control conditions were provided by delivering the same two stimulus sequences, but with the instruction of passively looking at the visual stimuli (passive tasks). The order of the two passive as well as of the two active tasks was counterbalanced across participants, but the passive tasks were delivered first at the beginning of the experimental session. In each task, 320 frequent stimuli were intermixed with 78–82 rare stimuli randomly distributed. Each task was split into two runs of approximately 7 min, followed by a 3 min rest. Altogether, eight stimulation runs (two passive and two active ones for either task) were administered during the experimental session. The total time inside the scanner was approximately 80 min.

### EEG Data acquisition and pre-processing

EEG was recorded with a MR-compatible amplifier (BrainAmp-MR, BrainProducts, Germany) placed inside the MR scanner and sampled at 5000 Hz. The amplifier was connected to the recording computer located outside the room by means of an optic cable. Subjects were fitted with a MR-compatible elastic cap (BrainCap-MR 32 Channel-Standard, BrainProducts, Germany) containing 31 electrodes (Fp1, Fp2, F7, F3, Fz, F4, F8, FC5, FC1, FC2, FC6, T7, C3, Cz, C4, T8, CP5, CP1, CP2, CP6, TP9, TP10, P7, P3, Pz, P4, P8, POz, O1, Oz, O2) and one electrode for electrocardiogram (ECG), that was placed on the chest. The impedances were kept below 5 kOhm. All the electrodes on the scalp were referenced to the common average reference. The EEG was analyzed with BrainVision Analyzer 2.2 software (Brainproducts, Germany). MRI artifacts were removed from EEG data using the Analyzer tool^58^. The EEG was then filtered from 0.01 Hz to 100 Hz and was also subjected to independent components analysis (ICA) to remove the ECG and eye-movement artifacts.

### ERP analysis

The EEG signal was segmented in epochs from −1200 ms to +1000 ms around stimulus onset and artifact-free epochs were averaged for each stimulus type (target/non-target), task (oddball/omitted target) and condition (active/passive). For the omitted target trials, the time 0 corresponded to 2000 ms following the preceding stimulus onset (i.e., the time at which a new stimulus would have appeared). The presence and latencies of ERP components for each experimental trial and stimulus type were determined on the maximal values of the Global Field Power (GFP) grand averaged over all subjects. The GFP is a reference-independent descriptor of the magnitude of the potential field over the scalp plotted as a function of time. It is calculated as the root summed square of the voltage of all recording electrodes simultaneously at each time point^59^. The GFP provides a useful summary of the ERP time-course. Statistical comparisons of the GFP were performed between target and non-target trials and between active and passive vision in both the Oddball and Omitted target tasks using running t-tests at each time point.

Pre-stimulus ERPs were analysed by averaging together frequent and target trials over the active tasks because differences between trials are not expected to occur in this interval (i.e., the stimulus type was unpredictable). In addition, incorporating more trials in the average provided a better signal-to-noise ratio. The mean amplitude of the first 200 ms of the ERP analysis epoch (−1200/−1000 ms) was taken as the baseline. Based on the GFP, ERP topography and previous studies (e.g.^35,56^), the last 500 ms of the pre-stimulus period were separated into an early phase (−500/−200 ms, maximal amplitude at lateral prefrontal scalp locations F7 and F8) and a late phase that included also the first 100 ms post-stimulus (−200/+100 ms, maximal at Fz and Cz). Statistical comparison of the mean amplitude of earlier activity was performed by 2 × 2 repeated measure analysis of variance (RM-ANOVA) with Task (oddball vs. omitted target) and Electrode (F7 vs. F8) as factors. In the later interval, another 2 × 2 RM-ANOVA was performed with Task and Electrode (Fz vs. Cz) as factors on the mean amplitude.

Post-stimulus ERP components were identified for the different tasks and their latencies and amplitudes were analysed. In order to exclude any possible effect of pre-stimulus differences between tasks, peak amplitudes were measured adopting a canonical −200/0 ms baseline just before the stimulus onset^3^. Grand average ERPs were obtained for target and non-target stimuli in the two tasks, and in both the active and the passive conditions. For statistical comparisons, a 2 × 2 RM-ANOVA with Task (oddball vs. omitted target) and Stimulus (target vs. non-target) as factors was executed on each component of the active tasks using the mean amplitude in the 20 ms interval around the peak.

To allow a direct comparison between ERP and fMRI analysis (see below) difference waves were also obtained. Specifically, the ERPs to rare stimuli in the passive tasks were subtracted from the target stimuli in the active tasks (i.e., oddball target *minus* passive rare; omitted target *minus* passive omitted rare): this procedure allowed extracting the ERP components specifically triggered by the target counting tasks.

### Dipole source modelling of ERP data

The topographical mapping of scalp voltage and the estimation of the intracranial sources of the ERP components in the grand-average waveforms were performed using Brain Electrical Source Analysis (BESA 2000 v.5.1.8; Megis Software GmbH, Gräfelfing, Germany). The algorithm implemented in BESA may estimate the location and orientation of multiple equivalent dipolar sources by calculating the scalp distribution obtained for a given dipole model (forward solution) and comparing it to the actual ERP distribution. Interactive changes in the location and orientation of the dipole sources lead to the minimization of the residual variance (RV) between the model and the observed spatiotemporal ERP distribution. The possibility of interacting dipoles was reduced via the selection of solutions with relatively low dipole moments with the aid of an “energy” constraint (weighted 20% in the compound cost function as opposed to 80% for the RV). The optimal parameter set was identified in an iterative manner by searching for a minimum in the compound cost function. In addition to the RV, the quality of the model was evaluated by applying residual orthogonality tests (ROTs; e.g.^60^). To model the dipolar sources, we used an fMRI-seeded strategy (for a similar approach see^35,36,57^), where the source location is not estimated by BESA, but from the fMRI data. Specifically, regions of interest were selected by clustering the fMRI spots, and the resulting coordinates (Table 1) were used to seed the sources. The source orientations were subsequently optimized to minimize the cross talk and interactions between the sources. To select the interval and the orientation order optimization (crucial to define the time course of the source), we followed the timing and the scalp topography of the ERPs. Modelling followed a sequential approach according to which the dipoles that accounted for the earlier portions of the waveform were maintained in place as additional dipoles were added. Thus, the number of dipoles chosen for these models corresponded to the major topographical features of the ERP waveforms. The rationale for this strategy was to use the fMRI information to solve the inverse problem of the ERP source localization.

**Table 1:** Latencies (ms) and amplitudes (*μV*) of grand-average post-stimulus ERP components for non-target and target stimuli in the active oddball and in the omitted-target tasks (np = not present).

	Oddball				Omitted-target			
	Non-Target		Target		Non-target		Target	
	Latency	Amplitude	Latency	Amplitude	Latency	Amplitude	Latency	Amplitude
**P1**	116	5.85	115	6.05	118	6.02	np	np
**pN1**	122	3.69	122	3.55	120	3.60	np	np
**N1**	193	2.74	195	276	200	3.18	np	np
**pP1**	195	1.46	194	1.96	192	1.55	np	np
**P2**	295	4.20	295	4.70	285	4.12	np	np
**pP2**	np	np	310	5.41	np	np	350	2.65
**P3**	np	np	450	5.98	np	np	520	3.92

### fMRI Data Acquisition and Pre-processing

The fMRI data were acquired using a Discovery MR 750, General Electric, Milwaukee, WI 3 Tesla scanner. fMRI data were obtained by a T2*-weighted gradient recalled echo-planar imaging sequence (TR 2000 ms, TE 40 ms, FA 90°, image matrix 128 × 128, in plane field of view 220 × 220 mm²) with 28 interleaved slices (slice thickness 4 mm, gap 1 mm) parallel to the anterior-posterior commissural plane. Acquisition was repeated over 226 volumes for a total scanning time of 7 min and 32 s. The same acquisition protocol was used for all the conditions (8 runs). For each subject, additional high-resolution T1-weighted images were acquired (three dimensional (3D)-BRAVO sequence, TR 8.5 ms, TI 450 ms, TE 3.3 ms, FA 12 degrees, voxel size 1 × 1 × 1 mm, 182 axial-oblique slices, total scanning time 4 min 42 s) to provide accurate anatomic references for functional data.

Data were analysed using the FEAT 6.00 tool of the software-package FSL 5.0.7 (www.fmrib.ox.ac.uk/fsl). Each functional dataset underwent preliminary processing including elimination of the first four volumes, slice-scan-time-correction, 3D motion-correction, high-pass temporal-filtering (100 s) and spatial-smoothing (Gaussian Kernel, 8 mm full-width-half-maximum). All subjects showed movement-related displacement lower than 3 mm/3 degrees.

Functional and structural datasets were aligned to Montreal Neurological Institute – 152 (MNI-152) standard space (International Consortium for Brain Mapping-152 [ICBM-152] template) by linear 12-degrees-of-freedom transformations (FLIRT tool). The subjects during acquisition wore earplugs with 27 dB attenuation, as well as “MRconfon” headphones (Magdeburg, Germany) providing further noise attenuation. The head of the subject was set at the isocenter of the magnet bore.

### fMRI analysis

Statistical analysis was performed by a general linear model. The regressors of interest (frequent stimuli and rare stimuli) were built by convolving the stimulus impulse function (amplitude of impulse = 200 ms) with the hemodynamic response function (Gamma function). Motion parameters were set as confounds. A fixed-effect model was adopted for high-level statistical analysis^61^. Two overlapped group statistical maps (Z-threshold = 3.3, cluster p-threshold < 0.05) were obtained comparing passive and active conditions for both standard oddball and omitted-target paradigm.

## Results

### Behavioral data

All the participants correctly reported the number of the targets with a high level of accuracy (hits > 98% in each of the active tasks), although all of them reported the omitted-target task as being more demanding.

### ERP data

**Figure 2:**
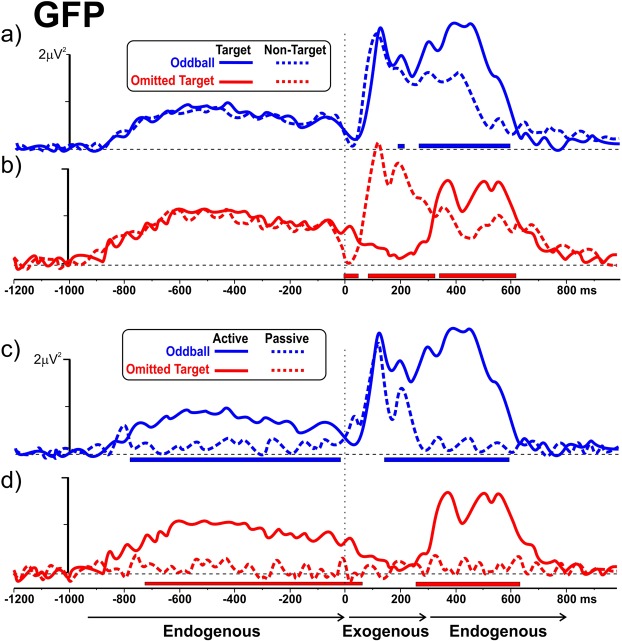
Global Field Power (GFP) of ERPs preceding and following the stimulus in the different experimental conditions. The GFP time course associated with Frequent/Non-Target and Rare/Target stimuli are presented for the Oddball Task (panel a, blue plot) and for the Omitted Target Task (panel b, red plot). GFP associated with Rare/Target stimuli in the passive and the active conditions are provided for the Oddball Task (panel c, blue plot) and Omitted Target Task (panel d, red plot). The horizontal thick blue and red lines signal epochs in which the difference between the GFP of the two ERPs was statistically significant. The temporal extents of Exogenous and Endogenous components are highlighted by the black thin horizontal arrows below the ERP traces.

Despite the difficult conditions of EEG recording within a strong magnetic field, a good and reliable ERP signal was obtained inside the scanner. The ERP GFP over the entire analysis epoch in the four experimental conditions allowed the identification of components preceding and following the stimuli and highlighted their exogenous or endogenous nature (Fig. 2). Exogenous components were elicited only within the first 300 ms post-stimulus, were absent whenever the expected stimulus was omitted. They reflected the bottom-up, pre-attentive processing of incoming –even task irrelevant- visual stimuli. Endogenous components, on the other hand, were observed both *preceding* and *following* the stimulus, their time-course spanning from −1000 ms to stimulus onset and extending, in the post-stimulus interval, from 300 ms on. They responded to both actual and omitted targets and were strictly contingent on the task instructions (different between target and non-target stimuli). Such endogenous signals expressed the engagement of a top-down attentional mechanism aimed at the detection of task-relevant stimuli. Figure 2 also shows the statistical comparison between the oddball and omitted target tasks, which is represented by the horizontal thick lines beneath the time axis. These lines indicate the interval with significant differences (p > 0.05) between GFP in the plotted tasks. Intervals with at least five contiguous points (20 ms) significantly different are displayed.

### Post-stimulus ERPs

**Figure 3:**
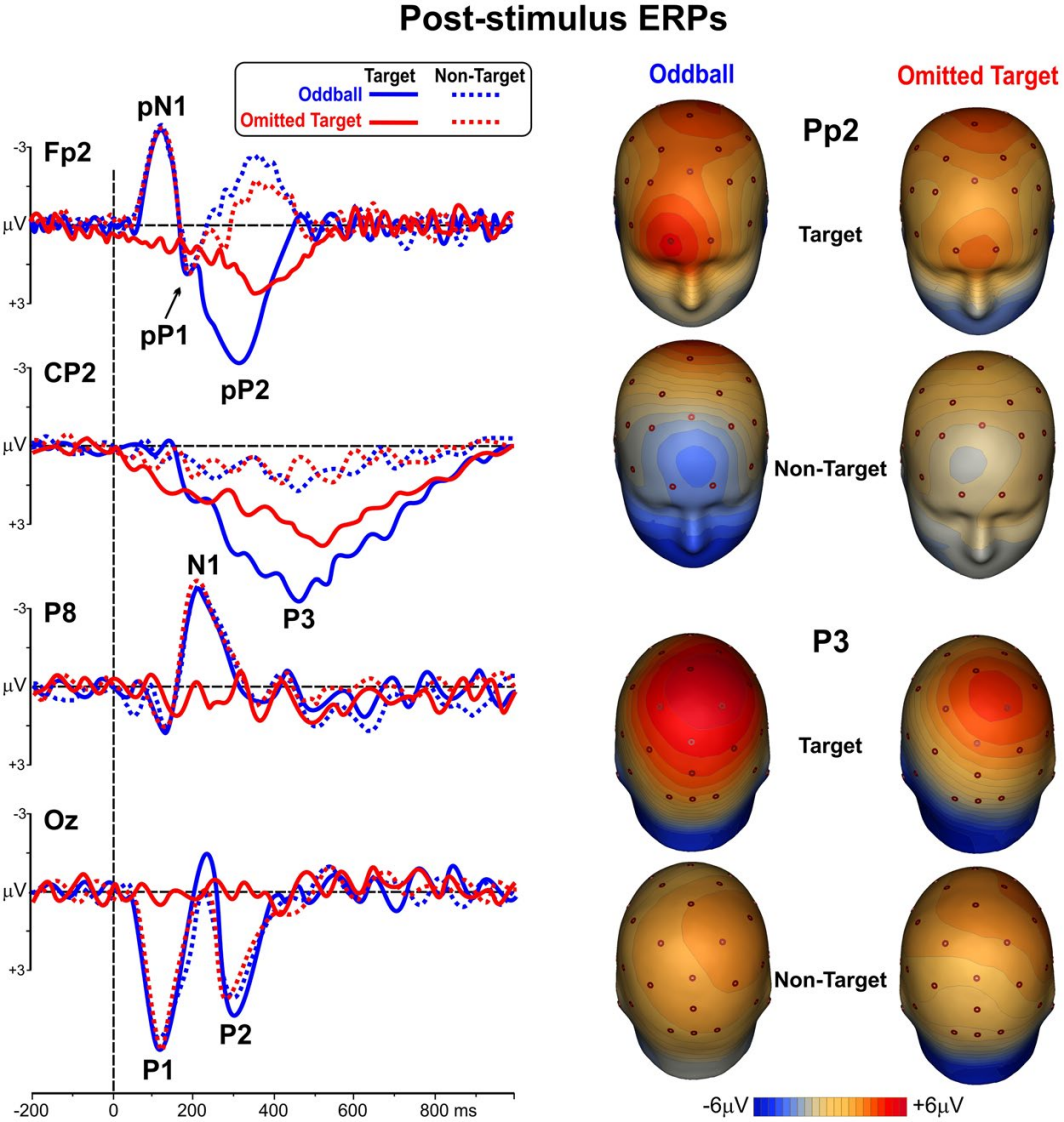
Left: ERP waveforms of the post-stimulus responses to non-target and target stimuli in the two active tasks. Right: scalp topography of the pP2 and P3b components.

Post-stimulus ERP waveforms for the two active tasks are shown in the left panel of Fig. 3. The positive P1 component peaked at 115 ms on medial occipital sites (see Oz). Two prefrontal components (pN1 and pP1) peaked at 120 ms and 190 ms respectively (see Fp2). The negative N1 peaked at 210 ms with a bilateral parieto-occipital distribution (see P8); the posterior P2 peaked at 295 ms at the Oz site. These components were present and comparable in all the experimental conditions, except in response to the target stimuli of the omitted target task: as expected, no sensory-related components emerged whenever the stimulus was missing. On the other hand, two longer-latency components were clearly detectable following all the target stimuli. Specifically, a pronounced positive peak was recorded over the prefrontal derivations (the pP2) larger and earlier (peaking at 300 ms) in the oddball task than in the omitted target task (peak at 350 ms). A centro-parietal P3 component was clearly detectable for target stimuli and peaked at 450 ms in the oddball task and at 520 ms (with a reduced amplitude) in the omitted target task. The right panel of Fig. 3 shows the scalp topography of the pP2 and the P3 components elicited by the target and non-target stimuli of the two tasks. RM-ANOVAs on the P1, pN1, N1, and P2 components showed that the main effects (Task and Stimulus) were not significant, but the interactions were significant (F_(1,12)_ > 14.8 p < 0.01). Bonferroni post-hoc comparison confirmed that all these early components were smaller (p < 0.01) for the omitted target stimuli (where they were actually absent) compared to the other conditions, which did not differ from each other. RM-ANOVAs on the pP2 and P3 showed significant effects of Task (F_(1,12)_ > 11.8 p < 0.006) and Stimulus (F_(1,12)_ > 13.5 p < 0.004) indicating that both components were larger in the oddball than in the omitted target task, and larger for target than non-target stimuli independently of the task. The interaction effects were not significant. An overview on the latencies and amplitudes of the post-stimulus ERP components is reported in Table 1.

### Pre-stimulus ERPs

**Figure 4:**
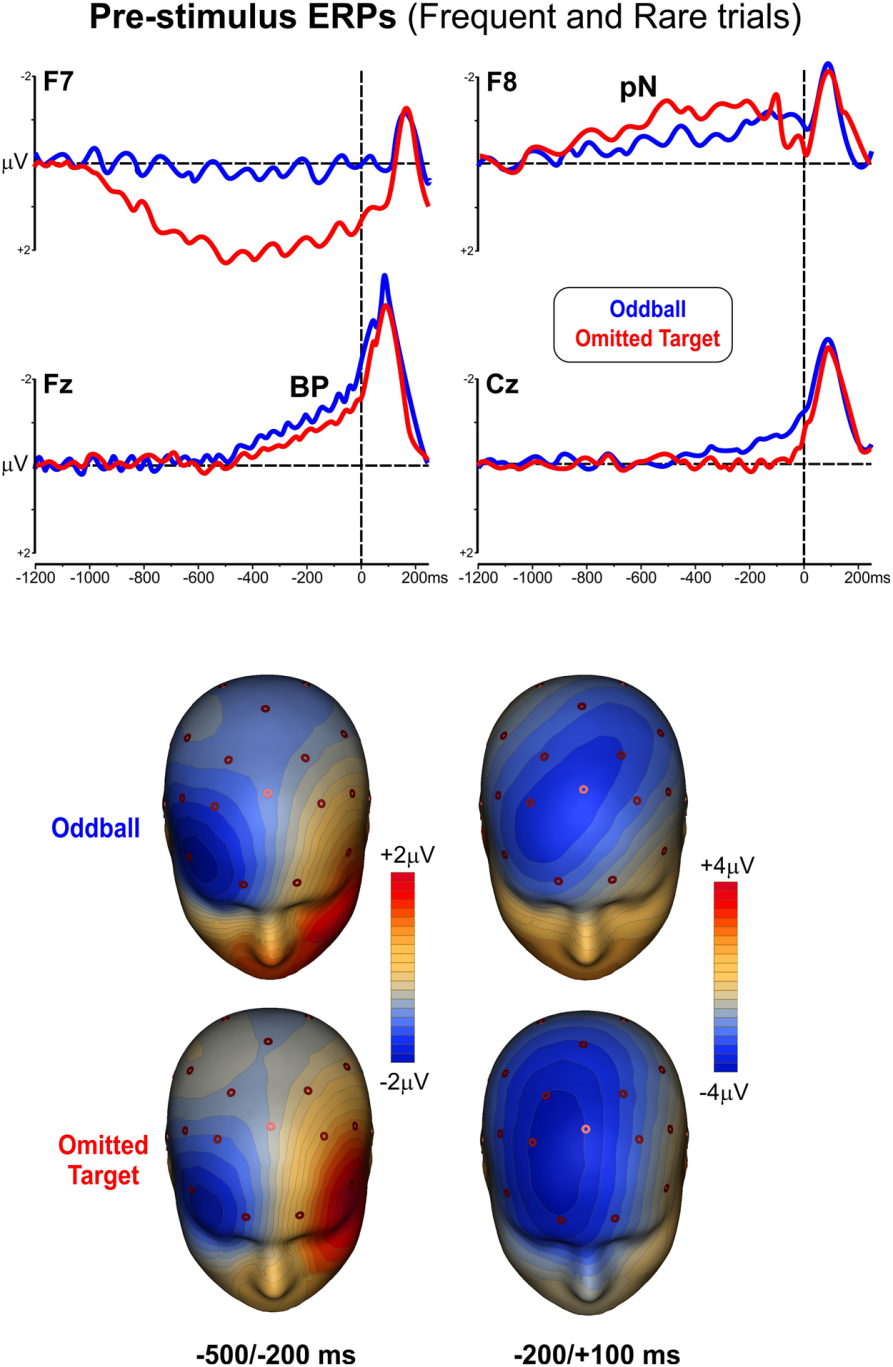
ERP waveforms (top) and scalp topography (bottom) of the pre-stimulus activities (pN, BP) in the two active tasks (oddball and omitted target). Maps represent the prefrontal lateral activity between −500/−200 ms (left) and the medial frontal activity between −200/+100 ms (right).

The top panel of Fig. 4 shows the pre-stimulus ERP waveforms obtained from averaging across both types of stimuli (non-target, target) in each active task. The lower panel of Fig. 4 shows the scalp topography of the early and late pre-stimulus intervals in the two tasks. Three major components were detected in the pre-stimulus period. A prefrontal slow rising negativity (prefrontal negativity, pN) was observed on the right lateral prefrontal scalp (electrode F8). This activity was quite similar in the two tasks, initiated very early around −900 ms and was maximum in the last 500 ms before the stimulus. Concomitantly with this negativity, in the active omitted-target task only, a slow positive shift was present on the left lateral prefrontal scalp (electrode F7) and reached its maximum amplitude around −500/−400 ms. To check-up for possible contamination of these slow waves by (horizontal) eye movements, in addition to ICA, a trial-by-trial examination of EEG signal over the frontal and anterior temporal electrodes was performed in each individual. Horizontal eye movements were rare (ranging from none to six over the entire recording session), balanced between left and right direction and produced no significant artefacts on the averaged ERPs. Starting from 500 ms before the stimulus onset, a negative slow component, steeper than pN, resembling a Bereitschaftspotential (BP) emerged on midline frontal sites on both active tasks. Statistical analysis on the earlier interval confirmed a significant Task x Electrode interaction (F_(1,12)_ = 11.49 p < 0.005). Post-hoc comparisons showed that the left prefrontal positivity on F7 was present in the omitted-target task only (p < 0.05), while the pN on F8 and the BP-like component on Fz and Cz did not differ between tasks and electrodes (all ps ns).

**Figure 5:**
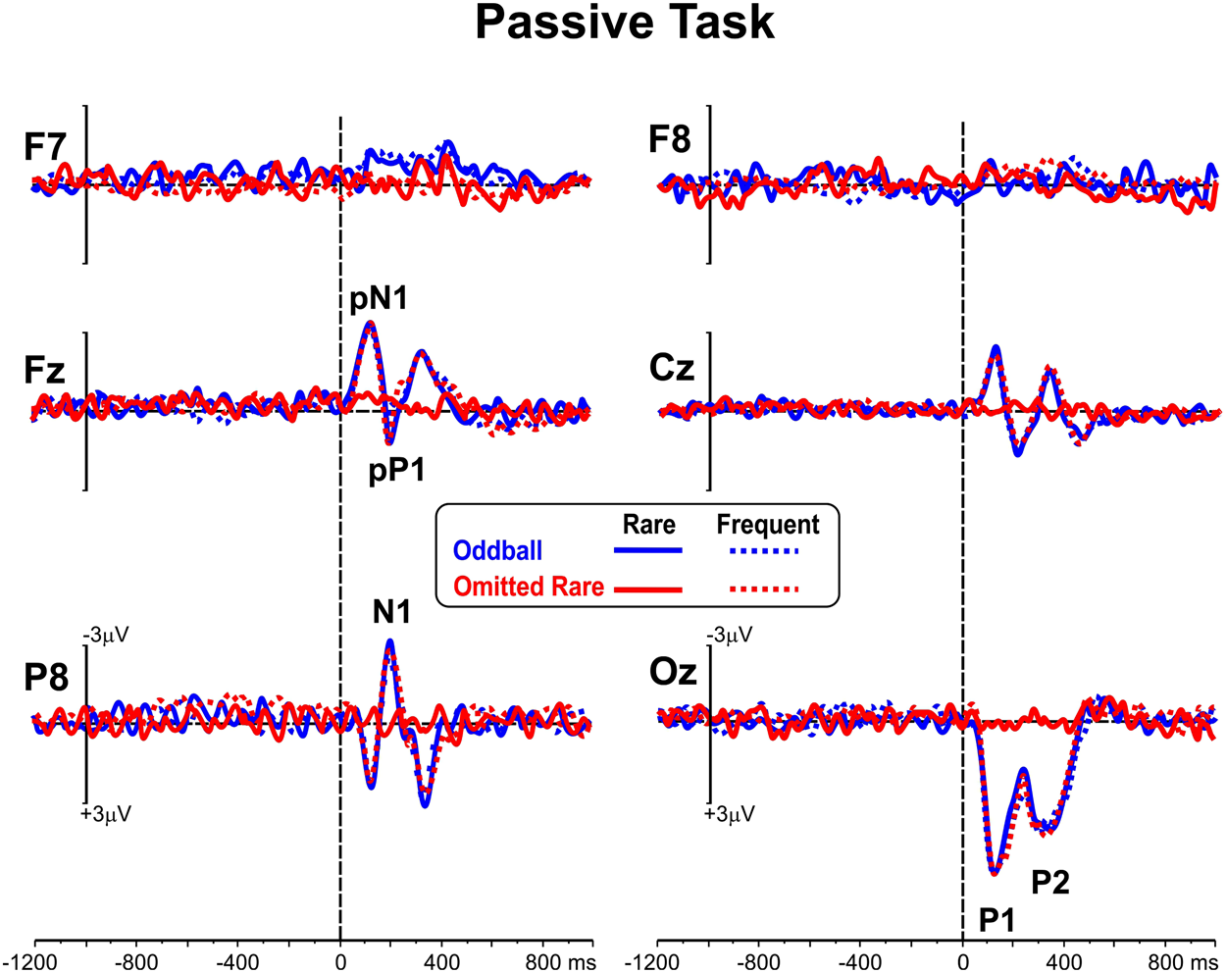
ERP waveforms in the two passive tasks for both frequent and rare stimuli.

Figure 5 shows the ERP waveforms recorded in the passive tasks. As can be noted, at variance with the active paradigms, no pre-stimulus slow waves were detected on the frontal sites, while the post-stimulus ERPs (i.e., P1, N1, P2, pN1, pP1) were comparable across tasks, except for the omitted-target stimuli, in which no component was recorded.

**Figure 6:**
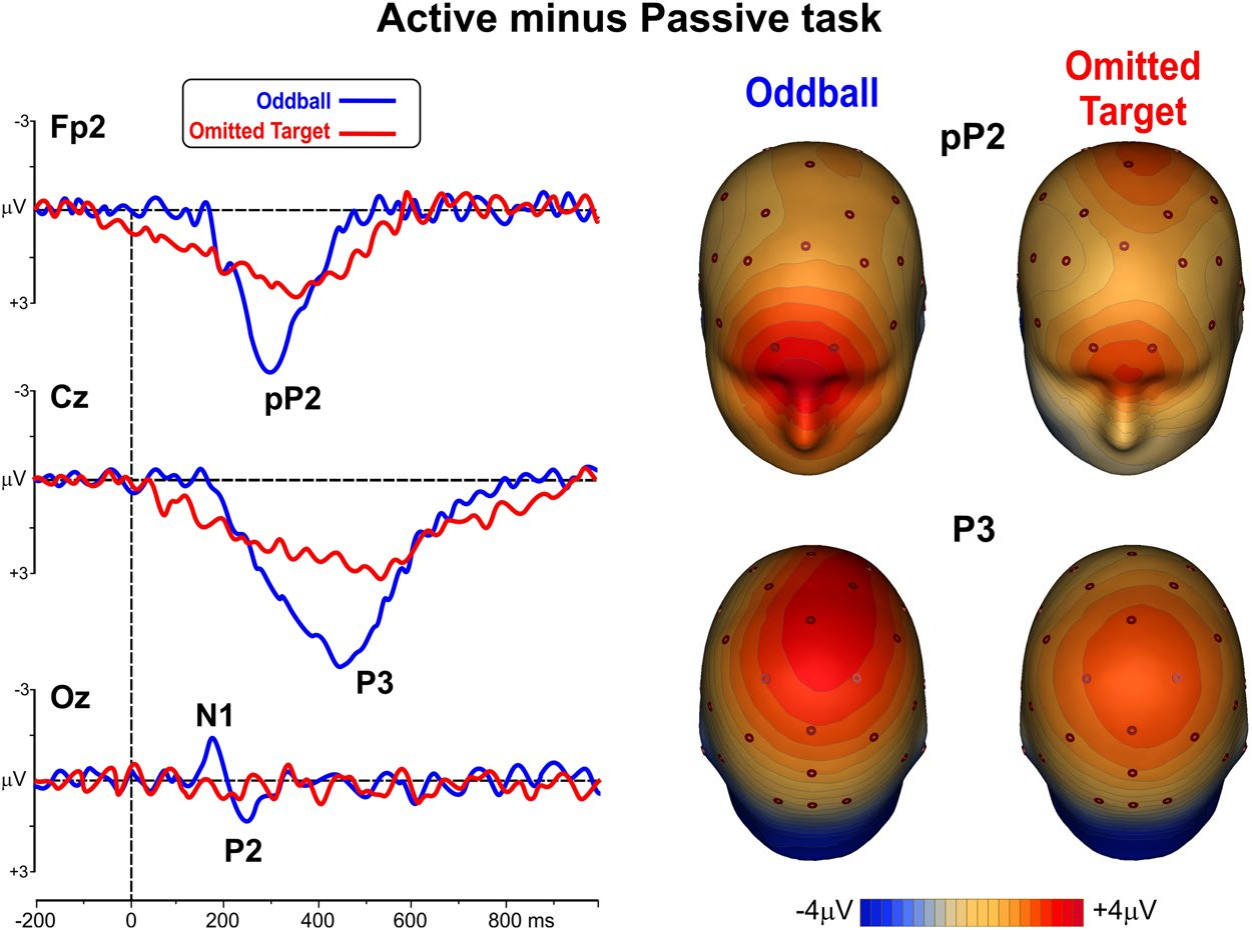
Left: post-stimulus ERP differential waveforms (active minus passive) following the rare stimuli. Right: scalp topographical distribution of the pP2 and P3 differential components.

In order to isolate the ERP activity specifically related to target identification, difference waves were calculated by subtracting ERPs to targets in the passive condition from the homologous responses obtained in the active condition, for each of the two tasks (oddball and omitted target). The differential pP2 and P3 waveforms and their scalp topography are shown in Fig. 6. ERP difference waveforms highlighted the pP2 and P3 target-related components and their medial prefrontal and centro-parietal voltage distribution. Moreover, in the oddball task the sensory components N1 and P2 were clearly present, reflecting the well-known focusing of the selective attention on the target stimuli.

Notably, the waveform to omitted targets showed an early positive slow drift over the frontal and centro-parietal locations (Figs 3 and 6) possibly representing a return to baseline of the pre-stimulus negativity. When the (target minus non-target) difference waveforms were obtained for the active omitted target task, this early slow shift disappeared (Supplementary Fig. 1) excluding its specificity to the processing of rare targets. We interpreted such an early onset of positivity as reflecting the increased trial-to-trial latency variability of the responses to missing stimuli, which resulted in smearing/broadening of the averaged ERP waveform as well as in flattening of its amplitude.

### fMRI Data

**Figure 7:**
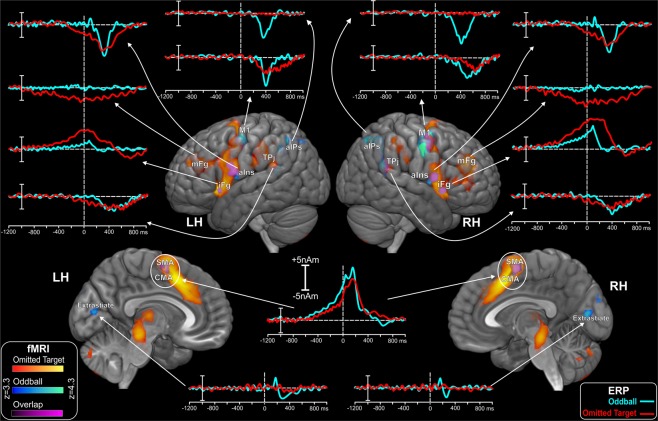
Spatiotemporal mapping obtained by the combination of ERP and fMRI data. fMRI activations resulting in the active > passive contrast for target stimuli for both tasks and their overlap from mesial and lateral views. LH, left hemisphere; RH, right hemisphere. Waveforms correspond to the ERP-based time-courses of the neural sources obtained from the fMRI-seeded dipoles. LH: left hemisphere. RH: right hemisphere. aIns: anterior Insula. aIPs: anterior IntraParietal sulcus. CMA: Cingulate Motor Area. iFg: inferior Frontal gyrus. mFg: middle Frontal gyrus. M1: motor cortex. SMA: Supplementary Motor Area. TPj: Temporal-Parietal junction.

fMRI results are shown on the brain templates of Fig. 7. The statistical map of the comparison between active and passive conditions is reported: the target trials with absent stimuli (omitted target) are rendered with red/yellow spots; the target trials with visual stimuli (oddball) are rendered in blue/cyan spots. The areas active for both condition (overlap) are rendered in purple. In the oddball condition, lateral activations were present in the inferior frontal gyrus (iFg), anterior insula (aIns), lateral premotor and motor cortex (M1), parietal cortex near the anterior intraparietal sulcus (aIPs) and around the temporal-parietal junction (TPj). Medially, activations were found in the supplementary and cingulate motor area (SMA and CMA) and in extrastriate occipital areas. In the omitted target task, fMRI showed activations in almost the same positions, but with a greater intensity and diffusion, and some crucial exceptions. The aIPs and the extrastriate areas were active in the oddball task only, and the motor cortex was more active in this task. Conversely, the middle frontal gyrus (mFg), deep cingulate regions, thalamus and cerebellum were active in the omitted target task only. The Talairach coordinates of the centre of gravity of the aforementioned areas are reported in Table 2. Besides these cortical activations, a significant BOLD response was observed in the midbrain and the central pons during the omitted target task but not during the standard oddball.

**Table 2:** Talairach coordinates (mm) of the local maxima of the regions activated during the oddball and the omitted-target tasks: the comparison is between the rare stimuli in the active and passive conditions for each task. *np*: not present.

ROIs		Oddball			Omitted target		
		x	y	z	x	y	z
**mFg**	LH		*np*		−28	42	22
	RH		*np*		36	44	20
**iFg**	LH	−40	12	−4	−38	16	−7
	RH	44	8	−8	43	12	−10
**SMA**	LH	−3	10	48	−2	17	32
	RH	2	13	44	1	16	47
**CMA**	LH	−6	18	10	−6	16	13
	RH	4	19	15	5	19	14
**aIns**	LH	−43	7	−2	−40	17	−3
	RH	44	16	1	43	15	−1
**Extrastriate**	LH	−12	−87	4		*np*	
	RH	10	−85	5		*np*	
**TPj**	LH	−57	−53	32	−51	−34	35
	RH	52	−39	30	53	−31	34
**aIPS**	LH	−46	−60	43	*np*		
	RH	40	−55	42	*np*		
**M1**	LH	−41	−3	40	−46	3	34
	RH	50	0	35	48	8	36

### ERP-fMRI Combination

In addition to the fMRI data, Fig. 7 reports the dipolar activities of the fMRI active regions for target stimuli in both the omitted target and oddball tasks. The reconstruction of the neural source activities was obtained by seeding the EEG dipoles on the fMRI activations (Table 2) and fitting the source orientation in successive time intervals representing the recorded ERP components. In the oddball task, the earliest activity started approximately 600 ms before stimulus onset with the slow rising negativity (pN) that obtained the lowest RV in the iFg identified in the fMRI experiment. The BP-like activity started approximately 550 ms before the stimulus: it was equally represented by the adjacent SMA and CMA and the resulting time-courses were very similar; for these reasons, they were represented by a single source. After stimulus onset, the N1 and P2 components were best represented by the activation in the medial extrastriate visual area. The pP2 component was associated with the activation in the bilateral anterior insula (aIns). The P3 was associated with the combined fMRI activations in the aiPs, TPj and lateral premotor and motor areas.

In the omitted target task, the earliest ERP activity initiated approximately 900 ms before stimulus onset with the slow rising positivity that obtained the lowest RV in the mFg. The pN component started at −800 ms and it was represented by the iFg. The BP-like shift started approximately 550 ms before the stimulus and it was represented by the adjacent SMA and CMA. The pP2 was associated with the activations in the bilateral anterior aIns whereas the P3 was associated with the combined fMRI activation in the TPj and lateral premotor and motor areas. These fMRI-seeded models explained 92.2% and 90.0% of the ERP variance in the time interval from −600 to 600 ms for the oddball task and the omitted target task, respectively.

## Discussion

The integration of ERPs with functional neuroimaging added value to our investigation, as the observed results were unobtainable with the use of the two techniques separately. This approach allowed overcoming the limitations of intracranial recordings (forcedly exploring only brain regions dictated by neurosurgical requirements) as well as the inconsistencies of lesion studies (arising from difficulties in disentangling the lesion local effect from the impact on distant neuronal networks). The results also provided some cues to highlight the functional role of the endogenous ERP components.

### Peculiarities of hemodynamic and electrophysiological activations

A first surprising result of our study was that the overall fMRI activity was greater and more widespread during the omitted target paradigm than during the standard oddball. This observation disproved our expectations that the greater the sensory stimulation (as during the visual target presentation) the stronger the BOLD response. However, the omitted target paradigm turned out being more demanding compared to the standard oddball task, as reported by our subjects at the end of the experimental sessions. In fact, it required greater attention and relied strongly on time estimation for promptly identifying the omitted stimuli. The activation of a fine perceptual timing mechanism was reflected in the fMRI responses elicited in the SMA, the thalamus and the cerebellum (Fig. 7), a set of brain structures well recognized as part of a cortico-subcortical network involved in metrical representation of time^62^. The time processing occurring here was of the type allowing the accurate estimation of relatively long intervals (2 s), an attention-dependent and elaborated mechanism mostly free from modality of stimulation^44^. This must be differentiated from the more elementary form of time processing, attention-independent, active when stimuli are presented at high frequency (>1 Hz). In addition, a higher level of arousal during the omitted target task was reflected by the peculiar hemodynamic activation observed in the rostral brainstem tegmentum, an expression of enhanced activity in the ascending reticular activating system^63^. Such a midbrain BOLD response was absent during the standard oddball paradigm.

The pattern of activations observed with fMRI (omitted target paradigm > oddball paradigm) was reversed with electrophysiological responses, namely the post-stimulus ERPs, as the late components (latencies > 300 ms) to target stimuli were of larger amplitudes in the oddball task than in the omitted target paradigm (Figs 2 and 3). This was not unexpected however, as the latency jitter of single responses to omitted targets is usually greater than that of responses to presented targets and this is reflected in the reduced amplitude of the averaged ERP^3,40,44^.

### Functional significance and localization of post-stimulus ERPs

The ERPs to target visual stimuli replicated our previous findings^35,56^. Multiple overlapping components with different polarity and topography were observed in the 100–300 ms latency range, reflecting the earlier sensory stages of visual processing (*exogenous* components elicited by “bottom up” visual input). Later ERP components in the post-stimulus interval were a prefrontal positivity (pP2, peaking at 300 ms) and the centro-parietal P3 wave (P3b or P300: latency 450 ms). When the omitted target paradigm was performed, only these two later components survived, albeit with reduced amplitudes and longer latencies due to the greater trial-to-trial jitter of the responses, a clear confirming of their *endogenous* nature. They reflect a “top-down” volitional engagement of cognitive processing set up by the task instructions and independent from the modality of the eliciting stimulus (they responded to an expected but omitted stimulus): therefore, they can be properly qualified as *emitted* potentials. The fact that scalp topographies as well as neural generators overlapped in the two experimental paradigms (oddball and omitted target) suggests that the same neuronal network underlies endogenous ERP components in both tasks.

The pP2 component in omitted target paradigms has never been reported before. Apparently, it is part of the stream of cognitive processing for the detection of salient events relevant for the current task: its role has been associated^64,65^ to accumulation of sensory evidence leading to target recognition and decision making (a process referred to as “perceptual decision making”^66^). For these reasons, the pP2 was also described as the correlate of the stimulus-response mapping process^67^. However, the present results reveal that sensory inputs are not necessary for eliciting a pP2, indicating that this component can be better described in terms of task-related categorization process, based on both external (e.g., stimulus) or internal (e.g., counting) events: what is necessary is just the task-relevance of those events in order to generate the pP2. Notably, in a recent study on the omission error (i.e., the missed response to the target stimulus presentation), two of us reported that the pN1 and pP1 components were preserved, but the pP2 was suppressed, suggesting that its absence was responsible for the failed process of stimulus categorization^68^. In the present ERP-fMRI study, the pP2 neural generators were localized in the bilateral anterior Insula confirming our previous investigations that recorded ERPs and fMRI in separate sessions^35,57^ as well as experiments based on ERPs^65,69^.

The Insula (Island of Reil), having multiple reciprocal connections with cortical and subcortical structures, is considered a supramodal center with integrative functions for multiple cerebral networks^70–72^. Anterior Insula has been recently proposed as a crucial node in a neural network supporting awareness^73,74^. Remarkably, the insula has been reported among the neural sources of P3 in most of the fMRI studies reported in the Introduction, although its physiological role has been rarely discussed. According to the present and previous studies, we conclude that the pP2 represents a crucial step in the neural circuitry underpinning the process of target recognition, strictly associated with, and preceding the P3, and possibly reflecting the earliest ERP sign of perceptual decision making and access of the stimulus to consciousness.

The pP2 must be differentiated from P3a or “novelty P3” for a number of reasons. First, its scalp topography is frontopolar, whereas the P3a shows a fronto-central distribution^5^. Second, being generated also when the sensory signal is omitted, the pP2 represents the product of a top-down attentional selection process driven by a higher order expectation system. In contrast, P3a is evoked by rare/distractor events, reflecting a bottom-up automatic attentional process engaged by intrusive stimuli: it is assumed being a correlate of the orienting response^5,75,76^. Third, the neural generators of pP2 and P3a are different. Our analysis confirmed the sources of pP2 in bilateral anterior insula (aIns), whereas a series of distributed generators has been described to contribute to P3a: fronto-temporo-parieto-cingular cortex, parahippocampus and the insula^14,19^. An ERP positive component (P2) quite similar to pP2 and different from P3a has been reported in another ERP-fMRI study using a visual oddball paradigm: it had a latency of 300 ms, a frontal scalp distribution and regional sources seeded in bilateral aIns^34^.

As for the P3 neural generators, the fMRI-guided ERP source analysis revealed that three pairs of bilateral fMRI activations were associated with this endogenous component. The temporo-parietal junction (TPj), the premotor and motor area (M1) and the anterior intraparietal sulcus (aIPs) combined their activity to produce the scalp P3 in the standard oddball task, whereas in the omitted target task the aIPs sources were absent. This aIPs activity, observed only when the target stimulus was delivered, probably reflects the contribution of a supramodal cortical area devoted to the analysis of sensory stimulation, as proposed by Di Russo *et al*.^35^. As for TPj, many fMRI studies have reported this important heteromodal association area as part of the neural network underpinning the P3^21,28,31^. Somehow unexpected was the identification of P3 sources in the bilateral premotor and motor areas since we deliberately used a covert counting task devoided of any motor performance to exclude contamination of P3 with movement-related activities. However, being that the premotor and motor cortices are “effector” areas, their activation suggests a link of P3 to response processing, independently from the modality of the response and the way the response is implemented. This finding partly contradicts the widely accepted interpretation of P3 as a reflection solely of the stimulus evaluation process^4,5^ and propose it as a correlate of the stimulus-driven response selection, leading to internal or external action^77^.

A negative result of our study was that no fMRI signals came from the medial temporal lobe (MTL: namely, the hippocampus), a brain structure frequently identified by intracerebral recordings as the principal P3 generator^14,15,78^. This negative result has been reported by the vast majority of fMRI studies, but no convincing explanation has been provided so far^22,34^. Crottaz-Herbette *et al*.^79^ blamed magnetic susceptibility artifacts leading to signal loss in the MTL region. Of note, simultaneous recordings of neuronal firing and ERPs from human MTL during an auditory oddball task showed unit suppression responses to target tones, suggesting an inhibitory synaptic mechanism underlying the MTL P3^80^. We propose that the absence of BOLD signal in the MTL during experiments with event-related fMRI might be explained by the reduced hemodynamic response associated with inhibition of hippocampal neurons elicited by the presentation of task-relevant stimuli. As the hippocampal P3 has a significantly longer latency than the scalp P3^15,81^, the suggestion is that the inhibition of MTL unit discharges is primarily produced by distant cortical neurons afferent to the medial limbic cortex.

### Functional significance and localization of pre-stimulus ERPs

Other noteworthy results came from the study of the slow pre-stimulus waves. These components belong to the class of anticipatory slow cortical potentials^6,55^ beginning as early as 1000 ms before the stimulus. The sustained negativity over the right prefrontal cortex can be identified as the prefrontal negativity (pN) whose sources are located in bilateral iFg, as already found by Di Russo *et al*.^35^. pN has been associated with prefrontal proactive inhibition coming in action to avoid unwanted responses during discriminative tasks^56^. The left prefrontal slow positive shift, observed only in the active omitted target task, was generated in the mFg and could represent an additional contribution of prefrontal control over a more challenging discriminative task. Finally, the neural sources of the mid-frontal negativity resembling BP were located bilaterally in the SMA and anterior cingulate. These midline areas are active in a number of cognitive tasks and have a role in response preparation and performance monitoring^54,82^. Therefore, the BP-like slow wave could reflect an anticipatory process essential for the optimal performance in a target detection paradigm. Further support to the above reported observations comes from the fact that no pre-stimulus ERPs were detected in the passive tasks, confirming that no engagement of prefrontal and/or premotor areas was required in absence of decisional requests.

### Differences from previous EEG-fMRI localization studies

The pattern of fMRI-guided ERP sources obtained in our study was partly at variance with the results of previous fMRI studies^22–25,28^ reporting more widespread mosaics of activations in cortical and subcortical regions, not aligned along a pre/post-stimulus timeline. This was due mainly to the procedure we applied for analyzing the data. On the one hand, by guiding ERP source modelling with anatomical constraints obtained from fMRI, we obtained a much more accurate and reliable spatiotemporal mapping of ERP generators, as this approach significantly reduces the infinite number of possible intracerebral sources identified by the inverse problem algorithm^34^. On the other hand, our strategy of measuring the ERP activity *preceding* the stimulus allowed to differentiate sources that were active in different and consecutive time-windows (pre/post-stimulus). Therefore, contrasting findings of previous studies might be explained by the fact that fMRI activations occurring *before* the stimulus were erroneously interpreted as underpinning *post-stimulus* ERP components, such as the P3.

### Limitations and perspectives

The acquisition and analysis of simultaneous EEG-fMRI signals are based on dedicated technology, demand qualified expertise and are time consuming. However, the peculiarity of our protocol is to disclose neuronal and hemodynamic activations associated with the omission effect, free from confounding bottom-up input. They are strictly contingent on the presence of attention and expectancy, demonstrating the active participation of the subject to the task in the absence of any motor response. It follows that the procedure can be used to track the anatomo-functional architecture of top-down neural processes subserving perception in normals as well as to detect covert cognition in patients with severe brain injury, such as those with prolonged disorders of consciousness (DoC: vegetative state, minimally conscious state^83^). Our protocol could contribute to detect command-following capacity in DoC patients, useful for rehabilitative efforts^84^.

## Conclusions

This study stems from the belief that an appropriate strategy for localizing the neural generators of task-related endogenous ERPs is using an omitted target paradigm. The data obtained confirmed this working hypothesis and offered some cues for the psychophysiological interpretations of endogenous ERPs.

We provide evidence for the possibility of recording *emitted* ERPs from omission of target stimuli in a simultaneous ERP-fMRI event-related paradigm. Such a procedure allowed the detailed spatiotemporal modeling of the neural generators of purely *endogenous* late potentials. The results of the omitted target oddball depicted an antero-to-posterior neural circuitry involved in the detection and processing of rare, task–relevant events. As for post-stimulus endogenous ERPs, bilateral anterior Insula contributed to the pP2 component, which emerged at 350 ms. The P3b was generated bilaterally in the frontal, temporal-parietal and parietal areas with a latency of 520 ms.

Anticipatory slow waves, also endogenous, were produced by anterior areas: namely, the pN and the positive slow wave originated from the inferior and middle frontal gyrus respectively, over the lateral brain surface, while the SMA-CMA areas over the medial cortex contributed to the BP-like component.

The simultaneous recording of hemodynamic and neural signals in an omitted target task explores the anatomo-functional architecture of purely endogenous cognitive processes.

## Supplementary information

Supplementary Figure 1

## References

[1] Sutton S, Tueting P, Zubin J, John ER (1967). Information delivery and the sensory evoked potential. Science.

[2] O’Connell RG, Dockree PM, Kelly SP (2012). A supramodal accumulation-to-bound signal that determines perceptual decisions in humans. Nature Neuroscience.

[3] Picton TW (2000). Guidelines for using human event-related potentials to study cognition. Psychophysiology.

[4] Duncan CC (2009). Event-related potentials in clinical research: Guidelines for eliciting, recording, and quantifying mismatch negativity, P300 and N400. Clin. Neurophysiol..

[5] Polich J (2007). Updating P300: an integrative theory of P3a and P3b. Clin. Neurophysiol..

[6] Van Boxtel GJM, Böcker KBE (2004). Cortical measures of anticipation. J. Psychophysiol..

[7] Näätänen R (2011). The mismatch negativity: an index of cognitive decline in neuropsychiatric and neurological diseases and in ageing. Brain.

[8] Salti M (2015). Distinct cortical codes and temporal dynamics for conscious and unconscious percepts. eLife.

[9] Sergent C, Baillet S, Dehaene S (2005). Timing of the brain events underlying access to consciousness during the attentional blink. Nature Neurosci..

[10] Koch C, Massimini M, Boly M, Tononi G (2016). Neural correlates of consciousness: progress and problems. Nature Rev. Neuroscience.

[11] Donchin E, Coles MGH (1988). Is the P300 component a manifestation of context updating?. Behavioral and Brain Sciences.

[12] Verleger R, Śmigasiewicz K (2016). Do rare stimuli evoke large P3s by being unexpected? A comparison of the oddball effects between standard-oddball and prediction-oddball tasks. Adv. Cogn. Psychol..

[13] Fell J (2004). Neural bases of cognitive ERPs: more than phase reset. J. Cogn. Neurosci..

[14] Halgren E, Marinkovic K, Chauvel P (1998). Generators of the late cognitive potentials in auditory and visual oddball tasks. Electroenceph. clin. Neurophysiol..

[15] McCarthy G, Wood CC, Williamson PD, Spencer DD (1989). Task-dependent field potentials in human hippocampal formation. J. Neurosci..

[16] Smith ME (1990). The intracranial topography of the P3 event-related potential elicited during auditory oddball. *Electroenceph*. clin. Neurophysiol..

[17] Knight RT (1984). Decreased response to novel stimuli after prefrontal lesions in humans. Electroenceph. clin. Neurophysiol..

[18] Nishitani N (1999). The role of hippocampus in auditory processing studied by event-related electric potentials and magnetic fields in epilepsy patients before and after temporal lobectomy. Brain.

[19] Bledowski C, Prvulovic D, Goebel R, Zanella FE, Linden DEJ (2004). Attentional systems in target and distractor processing: a combined ERP and fMRI study. NeuroImage.

[20] Clark VP, Fannon S, Lai S, Benson R, Bauer L (2000). Responses to rare visual target and distractor stimuli using event-related fMRI. J. Neurophysiol..

[21] Kiehl KA, Laurens KR, Duty TL, Forster BB, Liddle PF (2001). Neural sources involved in auditory target detection and novelty processing: an event-related fMRI study. Psychophysiology.

[22] Linden DEJ (1999). The functional neuroanatomy of target detection: an fMRI study of visual and auditory oddball tasks. Cerebral Cortex.

[23] McCarthy G, Luby M, Gore J, Goldman-Rakic P (1997). Infrequent events transiently activate human prefrontal and parietal cortex as measured by functional MRI. J. Neurophysiol..

[24] Menon V, Ford JM, Lim KO, Glover GH, Pfefferbaum A (1997). Combined event-related fMRI and EEG evidence for temporal-parietal cortex activation during target detection. NeuroReport.

[25] Opitz P, Mecklinger A, von Cramon DY, Kruggel F (1999). Combining electrophysiological and hemodynamic measures of the auditory oddball. Psychophysiology.

[26] Calhoun VD, Adali T, Pearlson GD, Kiehl KA (2006). Neural chronometry of target detection: fusion of hemodynamic and event-related potential data. NeuroImage.

[27] Mantini D, Corbetta M, Perrucci MG, Romani GL, Del Gratta C (2009). Large-scale brain networks account for sustained and transient activity during target detection. NeuroImage.

[28] Bénar CG (2007). Single-trial analysis of oddball-event-related potentials in simultaneous EEG-fMRI. Hum. Brain Mapp..

[29] Iannetti GD (2005). Simultaneous recording of laser-evoked brain potentials and continuous, high-field functional magnetic resonance imaging. NeuroImage.

[30] Liebenthal E (2003). Simultaneous ERP and fMRI of the auditory cortex in a passive oddball paradigm. NeuroImage.

[31] Mulert C (2004). Integration of fMRI and simultaneous EEG: toward a comprehensive understanding of localization and time-course of brain activity in target detection. NeuroImage.

[32] Otzenberger H, Gounot D, Foucher JR (2005). P300 recordings during event-related fMRI: a feasibility study. Cogn. Brain Res..

[33] Rusiniak M (2013). A modified oddball paradigm for investigations of neural correlates of attention: a simultaneous ERP-fMRI study. Magn. Reson. Mater. Phy..

[34] Bledowski C (2004). Localizing P300 generators in visual target and distractor processing: a combined Event-Related Potential and functional Magnetic Resonance Imaging study. J. Neurosci..

[35] Di Russo F (2016). Spatiotemporal brain mapping during preparation, perception, and action. NeuroImage.

[36] Pitzalis S, Strappini F, De Gasperis M, Bultrini A, Di Russo F (2012). Spatio-temporal brain mapping of motion-onset VEPs combined with fMRI and retinotopic maps. PlosOne.

[37] Gaillard AWK (1988). Problems and paradigms in ERP research. Biol. Psychol..

[38] Chennu S (2016). Silent expectations: Dynamic Causal Modeling of cortical prediction and attention to sounds that weren’t. J. Neurosci..

[39] Hernández OH, Vogel-Sprott M (2009). OSP parameters and the cognitive component of reaction time to a missing stimulus: linking brain and behavior. Brain & Cogn..

[40] Jongsma MLA (2005). Expectancy effects on omission evoked potentials in musicians and non-musicians. Psychophysiology.

[41] Klinke R, Fruhstorfer H, Finkenzeller P (1968). Evoked responses as a function of external and stored information. Electroenceph. clin. Neurophysiol..

[42] Perrault N, Picton TW (1984). Event-related potentials recorded from the scalp and nasopharynx. II. N2, P3 and slow wave. Electroenceph. clin. Neurophysiol..

[43] Picton TW, Hillyard SA (1974). Human auditory evoked potentials. II. Effects of attention. Electroenceph. clin. Neurophysiol..

[44] Renault, B. & Lesèvre, N. Topographical study of the emitted potential obtained after the omission of an expected visual stimulus, In *Multidiscipinary Perspectives in Event-related Brain Potential Research* (ed. Otto, D.) 200–208 (U.S. Govt. Printing Office Washington, D.C., 1978).

[45] Ritter W, Simson R, Vaughan HG, Friedman D (1979). A brain event related to the making of a sensory discrimination. Science.

[46] Ruchkin DS, Sutton S, Tueting P (1975). Emitted and evoked P300 potentials and variation in stimulus probability. Psychophysiology.

[47] Ruchkin DS, Sutton S (1978). Emitted P300 potentials and temporal uncertainty. Electroenceph. clin. Neurophysiol..

[48] SanMiguel I, Widmann A, Bendixen A, Trujillo-Barreto N, Schröger E (2013). Hearing silences: human auditory processing relies on preactivation of sound-specific brain activity patterns. J. Neurosci..

[49] Simson R, Vaughan HG, Ritter W (1976). The scalp topography of potentials associated with missing visual or auditory stimuli. Electroenceph. clin. Neurophysiol..

[50] Tarkka IM, Stokic DS (1998). Source localization of P300 from oddball, single stimulus and omitted stimulus paradigms. Brain Topogr..

[51] Wacongne C (2011). Evidence for a hierarchy of predictions and prediction errors in human cortex. Proc. Natl. Acad. Sci..

[52] Weinberg H, Walter GW, Crow HJ (1970). Intracerebral events in humans related to real and imaginary stimuli. Electroenceph. clin, Neurophysiol..

[53] Yabe H, Tervaniemi M, Reinikainen K, Näätänen R (1997). Temporal window of integration revealed by MMN to sound omission. NeuroReport.

[54] Shibasaki H, Hallett M (2006). What is the Bereitschaftspotential. Clin. Neurophysiol..

[55] Di Russo F (2017). Beyond the “Bereitschaftspotential”: Action preparation behind cognitive functions. Neuroscience and Biobehavioral Reviews.

[56] Berchicci M, Spinelli D, Di Russo F (2016). New insights into old waves by matching stimulus- and response-locked ERPs on the same time-window. Biol. Psychol..

[57] Sulpizio V (2017). Hemispheric asymmetries in the transition from action preparation to execution. NeuroImage.

[58] Allen PJ, Josephs O, Turner R (2000). A method for removing Imaging Artifact from continuous EEG recorded during functional MRI. Neuroimage.

[59] Lehmann D, Skrandies W (1980). Reference-free identification of components of checkerboard-evoked multichannel potential fields. Electroenceph. clin. Neurophysiol..

[60] Böcker KBE, Brunia CHM (1994). Van den Berg-Lenss, M.M.C. A spatio-temporal dipole model of the stimulus preceding negativity SPN prior to feedback stimuli. Brain Topogr..

[61] Beckmann CF, Smith S, Jenkinson M (2003). General multi-level linear modelling for group analysis in fMRI. Neuroimage.

[62] Coull JT, Cheng R-K, Meck WH (2011). Neuroanatomical and neurochemical substrates of timing. Neuropsychopharmacol. Rev..

[63] Kinomura S, Larsson J, Gulyás B, Roland PE (1996). Activation by attention of the human reticular formation and thalamic intralaminar nuclei. Science.

[64] Darriba A, Waszak F (2018). Predictions through evidence accumulation over time. Sci. Rep..

[65] Perri RL (2018). Awareness of perception and sensory-motor integration: ERPs from the anterior insula. Brain Struct. Funct..

[66] Heekeren HR, Marrett S, Ungerleider LG (2008). The neural systems that mediate human perceptual decision-making. Nat. Rev. Neurosci..

[67] Perri RL, Berchicci M, Lucci G, Spinelli D, Di Russo F (2015). Why do we make mistakes? Neurocognitive processes during the preparation–perception–action cycle and error-detection. Neuroimage.

[68] Perri RL, Spinelli D, Di Russo F (2017). Missing the target: the neural processing underlying the omission error. Brain Topogr..

[69] Perri RL, Berchicci M, Bianco V, Spinelli D, Di Russo F (2018). Brain waves from an “isolated” cortex: Contribution of the anterior insula to cognitive functions. Brain Struct. Funct..

[70] Augustine JR (1996). Circuitry and functional aspects of the insular lobe in primates including humans. Brain Res. Rev..

[71] Cauda F (2011). Functional connectivity of the insula in the resting brain. NeuroImage.

[72] Uddin LQ, Nomi JS, Hébert-Seropian B, Ghaziri J, Boucher O (2017). Structure and function of the human Insula. J. Clin. Neurophysiol..

[73] Craig ADB (2009). How do you feel - now? The anterior insula and human awareness. Nat. Rev. Neurosci..

[74] Fischer DB (2016). A human brain network derived from coma-causing brainstem lesions. Neurology.

[75] Courchesne E, Hillyard SA, Galambos R (1975). Stimulus novelty, task relevance and the visual evoked potential in man. Electroenceph. clin. Neurophysiol..

[76] Spencer KM, Dien J, Donchin E (2001). Spatiotemporal analysis of the late ERP responses to deviant stimuli. Psychophysiology.

[77] Verleger R, Baur N, Metzner MF, Śmigasiewicz K (2014). The hard oddball: effects of difficult response selection on stimulus-related P3 and on response-related negative potentials. Psychophysiology.

[78] Brázdil M, Roman R, Daniel P, Rektor I (2003). Intracerebral somatosensory event-related potentials: effect of response type (button pressing versus mental counting) on P3-like potentials within the human brain. Clin. Neurophysiol..

[79] Crottaz-Herbette S, Lau KM, Glover GH, Menon V (2005). Hippocampal involvement in detection of deviant auditory and visual stimuli. Hippocampus.

[80] Heit G, Smith ME, Halgren E (1990). Neuronal activity in the human medial temporal lobe during recognition memory. Brain.

[81] Halgren E (1995). Intracerebral potentials to rare target and distractor auditory and visual stimuli. II. Medial, lateral and posterior temporal lobe. Electroenceph. clin. Neurophysiol..

[82] Carter CS (1998). Anterior cingulate cortex, error detection and the online monitoring of performance. Science.

[83] Ragazzoni A (2017). Clinical neurophysiology of prolonged disorders of consciousness: from diagnostic stimulation to therapeutic neuromodulation. Clin. Neurophysiol..

[84] Curley WH, Forgacs PB, Voss HU, Conte MM, Schiff ND (2018). Characterization of EEG signals revealing covert cognition in the injured brain. Brain.

