# Supplementary material for: “Hit the missing stimulus”. A simultaneous EEG-fMRI study to localize the generators of endogenous ERPs in an omitted target paradigm
Source: Sci Rep. 2019 Mar 6;9:3684. doi: 10.1038/s41598-019-39812-z (PMC6403295; doi:10.1038/s41598-019-39812-z)
Supplement: Supplementary file 1 — Supplementary Figure 1 [file 41598_2019_39812_MOESM1_ESM.pdf]

“Hit the missing stimulus”. A simultaneous EEG-fMRI study to localize the generators of endogenous ERPs in an omitted target paradigm

Aldo Ragazzoni [a], Francesco Di Russo [b c*], Serena Fabbri [d e], Ilaria Pesaresi [d], Andrea Di Rollo [f], Rinaldo Livio Perri [b], Davide Barloscio [f], Tommaso Bocci [f,h], Mirco Cosottini [d e,] Ferdinando Sartucci [f g]

[a]PAS Foundation, Scandicci, [b]Department of Movement, Human and Health Sciences, University of Rome “Foro Italico”, Rome, [c]Santa Lucia Foundation (IRCCS Fondazione Santa Lucia), Rome, [d]Neuroradiology Unit, A.O.U.P., Pisa, [e]Department of Translational Research and New Technologies in Medicine and Surgery, University of Pisa, Pisa [f]Department of Clinical and Experimental Medicine, Unit of Neurophysiopathology, Pisa University Medical School, Pisa, [g]CNR, Neuroscience Institute, Pisa; Italy, [h] "Aldo Ravelli" Research Center, Department of Health Sciences, University of Milan & Ospedale San Paolo, Milan, Italy.

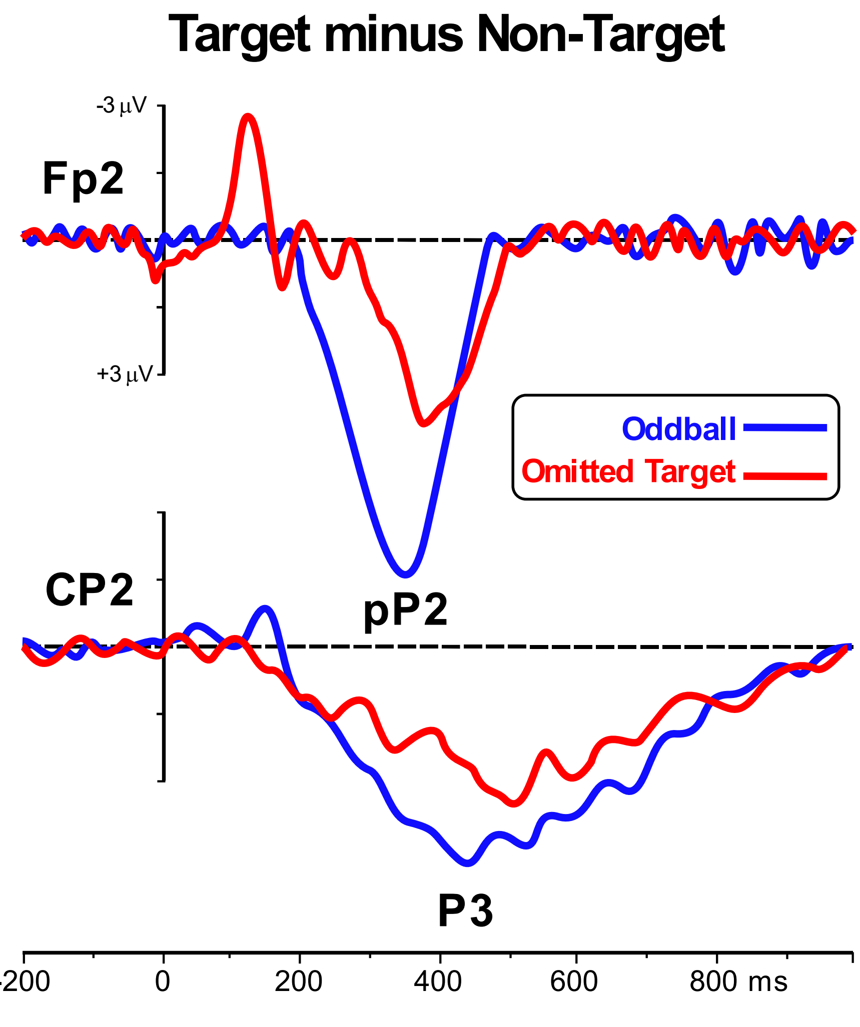

Supplementary Figure 1: Post-stimulus ERP difference (target minus non-target) waveforms obtained in the two active tasks (oddball and omitted-target) over the right prefrontal (Fp2) and centro-parietal (CP2) electrodes.
